# Tacrolimus pharmacokinetics in pediatric nephrotic syndrome: A combination of population pharmacokinetic modelling and machine learning approaches to improve individual prediction

**DOI:** 10.3389/fphar.2022.942129

**Published:** 2022-11-15

**Authors:** Qiongbo Huang, Xiaobin Lin, Yang Wang, Xiujuan Chen, Wei Zheng, Xiaoli Zhong, Dewei Shang, Min Huang, Xia Gao, Hui Deng, Jiali Li, Fangling Zeng, Xiaolan Mo

**Affiliations:** ^1^ Department of Pharmacy, Guangzhou Women and Children’s Medical Center, Guangzhou Medical University, Guangzhou, China; ^2^ Department of Pharmacy, The First Affiliated Hospital of Sun Yat-sen University, Guangzhou, China; ^3^ Department of Clinical Pharmacy, Wuhan Children’s Hospital (Wuhan Maternal and Child Healthcare Hospital), Tongji Medical College, Huazhong University of Science and Technology, Wuhan, China; ^4^ Department of Medical Big Data Center, Guangdong Provincial People’s Hospital, Guangdong Academy of Medical Sciences, Guangzhou, China; ^5^ Institute of Clinical Pharmacology, School of Pharmaceutical Sciences, Sun Yat-sen University, Guangzhou, China; ^6^ Department of Pharmacy, The Affiliated Brain Hospital of Guangzhou Medical University, Guangzhou, China; ^7^ Division of Nephrology, Guangzhou Women and Children’s Medical Center, Guangzhou Medical University, Guangzhou, China

**Keywords:** tacrolimus, pediatric nephrotic syndrome, population pharmacokinetic, machine learning, gene polymorphisms

## Abstract

**Background and Aim:** Tacrolimus (TAC) is a first-line immunosuppressant for the treatment of refractory nephrotic syndrome (RNS), but the pharmacokinetics of TAC varies widely among individuals, and there is still no accurate model to predict the pharmacokinetics of TAC in RNS. Therefore, this study aimed to combine population pharmacokinetic (PPK) model and machine learning algorithms to develop a simple and accurate prediction model for TAC.

**Methods:** 139 children with RNS from August 2013 to December 2018 were included, and blood samples of TAC trough and partial peak concentrations were collected. The blood concentration of TAC was determined by enzyme immunoassay; *CYP3A5* was genotyped by polymerase chain reaction-restriction fragment length polymorphism method; *MYH9*, *LAMB2*, *ACTN4* and other genotypes were determined by MALDI-TOF MS method; PPK model was established by nonlinear mixed-effects method. Based on this, six machine learning algorithms, including eXtreme Gradient Boosting (XGBoost), Random Forest (RF), Extra-Trees, Gradient Boosting Decision Tree (GBDT), Adaptive boosting (AdaBoost) and Lasso, were used to establish the machine learning model of TAC clearance.

**Results:** A one-compartment model of first-order absorption and elimination adequately described the pharmacokinetics of TAC. Age, co-administration of Wuzhi capsules, *CYP3A5* *3/*3 genotype and *CTLA4* rs4553808 genotype were significantly affecting the clearance of TAC. Among the six machine learning models, the Lasso algorithm model performed the best (R^2^ = 0.42).

**Conclusion:** For the first time, a clearance prediction model of TAC in pediatric patients with RNS was established using PPK combined with machine learning, by which the individual clearance of TAC can be predicted more accurately, and the initial dose of administration can be optimized to achieve the goal of individualized treatment.

## Introduction

Nephrotic syndrome is a disease characterized by massive proteinuria, hypoproteinemia, edema and hyperlipidemia as the main clinical manifestations, and is one of the most common glomerular diseases in children (Noone et al., 2018). Currently, tacrolimus (TAC), a calcineurin inhibitor, is the first-line immunosuppressive agent for the treatment of pediatric refractory nephrotic syndrome (RNS). However, the narrow therapeutic window and large individual differences in pharmacokinetics of TAC require adjustment of individual dosing by therapeutic drug monitoring (TDM), yet TDM reflects changes in blood concentrations with a lag. Therefore, it is important to identify the significant factors affecting the pharmacokinetic parameters of TAC and establish a prediction model before dosing to achieve individualized treatment.

Large individual differences in the pharmacokinetics of TAC are mainly due to clinical characteristics and genetic polymorphisms of patients. Studies have shown that clinical factors such as body weight, age and combined use of Wuzhi capsules significantly influence TAC pharmacokinetic parameters (Hao et al., 2018; Wang D. et al., 2019a; Wang X. et al., 2019b; Chen et al., 2020; Huang et al., 2020). Genetic factors include genetic polymorphisms related to metabolic enzymes, transporter proteins, receptors and other drug targets (Evans and Relling, 1999). Currently, *CYP3A5* genotype is the only key factor that has been shown to affect TAC pharmacokinetics (Birdwell et al., 2015). However, *CYP3A5* genotype does not fully reflect the individual differences in TAC pharmacokinetics. Other genetic factors include the renal podocyte-associated genes *ACTN4* and *MYH9* (Pecci et al., 2018; Feng et al., 2020), the pharmacodynamic pathway protein gene *MAP3K*, the *CTLA4* gene associated with the immune response, and inflammatory cytokine genes such as *IL2RA* may also influence the pharmacokinetics of TAC (Agrawal et al., 2020). In addition, most established PPK models in children with nephrotic syndrome incorporate only *CYP3A5* as a genetic variable, with limited studies of other relevant genes. Therefore, it is necessary to investigate the clinical characteristics and the comprehensive genetic variables when we build models to predict TAC pharmacokinetics.

Currently, methods for predicting pharmacokinetics in different individuals mainly include traditional multiple linear regression methods, population pharmacokinetic (PPK) methods, and machine learning methods (Agrawal et al., 2020; Li et al., 2021; Woillard et al., 2021). Multiple linear regression methods are simple to operate, but they cannot distinguish inter-and intra-individual variability. In contrast, PPK can fully account for inter-and intra-individual variation, but requires collecting multiple blood points for perfect model performance (Bon et al., 2018; Campagne et al., 2019). Unlike traditional statistical methods and PPK methods, machine learning methods are capable of handling complex, multidimensional data. Based on the characteristics of these various methods, Tang *et al.* developed a prediction model for six renal clearance drugs by combining PPK with machine learning methods (Tang et al., 2021), validating the good performance of novel approach of combining PPK with machine learning. Nevertheless, it is worth mentioning that there is no model for predicting TAC pharmacokinetics using PPK combined with machine learning.

Therefore, the purpose of this study was first to investigate the effects of clinical characteristics and genetic polymorphisms on the pharmacokinetics of TAC in pediatric patients with nephrotic syndrome, and to develop a PPK model for this population. Further, a simpler and more accurate machine learning model for predicting the clearance of TAC was developed based on the above PPK model, to provide a powerful tool for individualized therapy of TAC in pediatric patients with nephrotic syndrome.

## Methods

### Patients and sample collection

Patients diagnosed with nephrotic syndrome and taking oral TAC ((Prograg™, Astellas, Killorglin, Ireland) at Guangzhou Women and Children’s Medical Center between August 2013 and December 2018 were included in this study. Inclusion criteria: (1) All children were diagnosed in accordance with the 2012 KDIGO guidelines for the diagnosis of nephrotic syndrome (Lombel et al., 2013). (2) Steroid resistance or dependence. (3) Age of onset was less than 18 years. (4) Regularly taking TAC (Prograg™, Astellas, Killorglin, Ireland) and steroid (prednisone, prednisolone) as prescribed; Exclusion criteria: (1) Those who cannot take the medication regularly and follow up. (2) Combined use of other immunosuppressive agents (e.g. cyclophosphamide). (3) Combined use of drugs affecting the blood concentration of TAC (e.g. verapamil, ketoconazole, itraconazole). (4) Those who are allergic to macrolides. (5) Combination of liver and renal impairment and other disorders.

The starting dose of TAC in the dosing regimen was 500–3000 μg, administered orally every 12 h, and the dose was adjusted according to the whole blood trough concentration (target concentration 5–10 μg L^−1^). 2 ml of peripheral venous blood was collected in ethylenediaminetetraacetic acid (EDTA) anticoagulation tubes from children half an hour before dosing in the early morning, and blood samples at peak concentration were also collected from some children after dosing (0.5∼3 h). Clinical data (gender, age, weight, daily dose of TAC, records of co-administered drugs, laboratory test results, *etc*) were collected from the children. The whole blood concentration of TAC was determined by enzyme multiplication immunoassay technique (Viva-E, Siemens, Germany).

### DNA extraction and genotyping

DNA was extracted using the Genomic Tiangen Blood Deoxyribonucleic Acid Extraction Kit (DP348, Beijing, China). Polymerase chain reaction-restriction fragment length polymorphism method was used to determine *CYP3A5* rs776746, and MALDI-TOF MS method (Agena Bioscience MassARRAY^®^ system [Agena Bioscience, San Diego, CA, United States)] was used to detect other SNPs. The Hardy-Weinberg equilibrium test was performed using the chi-square test.

### Data analysis software

SPSS software (version 25.0) was used for univariate analysis; Phoenix NLME pharmacokinetic software (Version 8.1, Pharsight Corporation, United States) was used for PPK modelling; Scikit-learn 0.19.1 plug-in package in Python 3.6.5 was used for machine learning modelling.

### Population pharmacokinetic modelling

Pharmacokinetic data of TAC were analyzed for PPK using a nonlinear mixed effects model (NLME) approach. First-order conditional estimation-extended least squares (FOCE-ELS) was applied to all model runs.

### Base model

A one-compartment model of first-order absorption and elimination was used to fit the data. The absorption rate constant (Ka) was fixed at 4.48 h^−1^ based on reports in the literature and suitability for pediatric populations (Wang D. et al., 2019a; Chen et al., 2020; Huang et al., 2020; Li et al., 2021). Inter-individual variation was described using an exponential model.

$$P_{i}=TV\left(P\right)\times \exp\left(\eta_{i}\right);\;\eta_{i}∼N\left(0,\;\omega_{p}^{2}\right)$$

(where *P*_*i*_ is the value of the pharmacokinetic parameter for the ith subject, TV(*P*) is the population typical value of this parameter, and η_i_ is the interindividual random variation obeying a normal distribution with mean 0 and variance $\omega_{p}^{2}$ )

The following model was used to assess the residual variance.

$$Additive\;model:Y_{obs}=Y_{pred}+\varepsilon$$

$$Proportional\;model:\;Y_{obs}=Y_{pred}\times \left(1+{\;\varepsilon }_{1}\;\right)$$

$$Combined\;model:\;Y_{obs}=Y_{pred}\times \left(1+{\;\varepsilon }_{1}\;\right)+{\;\varepsilon }_{2}$$

$$Exponential\;model:\;Y_{obs}=Y_{pred}\times \exp\left(\varepsilon \right)$$

(where Y_obs_ is the measured concentration, Y_pred_ is the model prediction, and ε is the residual variance obeying a normal distribution with mean 0 and variance σ^2^)

The optimal residual variance model was determined using -2 times the logarithm of the maximum likelihood (−2 log likelihood, −2LL), the standard value of the Akaike information criterion (AIC), and the goodness-of-fit plot (GOF)).

### Covariate screening

The variables screened included: gender, age, weight, erythrocyte pressure (HCT), serum albumin (ALB), *etc,* prior to administration, and the combination of Wuzhi capluses (combWZ) was considered in the combined dosing. Gene-related factors were included in *CYP3A5* rs776746, *MYH9* rs2239781, *LAMB2* rs62119873, *ACTN4* rs62121818, *ACTN4* rs3745859, *MAP3K11* rs7946115, *ACTN4* rs56113315, *MYH9* rs4821478, *INF2* rs1128880, *IL2RA* rs12722489, *NPHS2* rs2274622, *CTLA4* rs4553808, *etc*. These SNPs were all variables that were correlated with TAC concentrations after a prior univariate analysis (*p* < 0.1) (Mo et al., 2020).

In this study, the stepwise regression method was used to screen covariates, and the selection of covariates was determined by the objective function value (OFV). In the forward selection process, covariates with an OFV decrease >3.84 (*p* < 0.05) were included in the model. In the backward elimination process, covariates with an OFV increase >6.63 (*p* < 0.01) were retained in the model. The principle follows the extended least square (ELS) method, where OFV is the -2LL value in the fitting process, and the smallest value of -2LL is used as the overall measure of the fitting effect. The expressions for the continuous and categorical covariate models are as follows, respectively.

$$P_{i}=TV\left(P\right)\times {\left(\frac{COV}{{COV}_{median}}\right)}^{\theta }$$

$$P_{i}=TV\left(P\right)\times \exp\left(\theta COV\right)$$

(COV: value of covariate; θ: fixed parameter coefficient of covariate; COV_median_: median of covariate)

Model evaluation and validation (1) Plot the goodness-of-fit (GOF) of the base model and the final model for visual evaluation, including: ① population predicted value-detected value (PRED-DV), ② individual predicted value-measured value (IPRED-DV), ③ conditional weight residual-population predicted value (CWRES-PRED), and ④ conditional weight residual-time (CWRES-TIME) of the scatter plot. (2) The final model is validated using visual predictive check (VPC). (3) Bootstrap method based on data resampling technique is used for internal validation of the final model. (4) Normalized prediction distribution error (NPDE) is used to evaluate the predictive performance of the final model based on a Monte Carlo simulation with the R software (Version 4.1.0,http://www.r-project.org). NPDE results are summarized graphically using: ① quantile-quantile plot of the NPDE, ② a histogram of the NPDE, ③ scatterplot of NPDE vs. time, and ④ scatterplot of NPDE vs. PRED.

## Machine learning modelling

### Data pre-processing

Machine learning modelling was performed on the variables screened by PPK. The categorical variables were first discretized.

### Data modelling

The data set is randomly divided into a test set and a training set by 3:7. Based on the five-fold cross-validation and hyperparameter search, the training set is used for the model generation: the training set is randomly divided into five equal parts, and the leave-one method is used. Each time, one piece of data is left for model verification, and the remaining four pieces of data are searched for hyperparameters to build a model. This is repeated 5 times, and the best model hyperparameters combination is finally determined. Furthermore, the five data sets are used to build a model based on a combination of certain hyperparameters, and the test set is used to evaluate the model. In this step, the hyperparameters will be fine-tuned. Then, re-divide the data set randomly into the test set and the training set by 3:7, repeat the above modelling process, and loop 5 times in this way. Finally, each algorithm will get 5 models, and the average of the performance of the 5 models is taken as the final model performance result.

## Results

### Patient demographic characteristics

**FIGURE 1:**
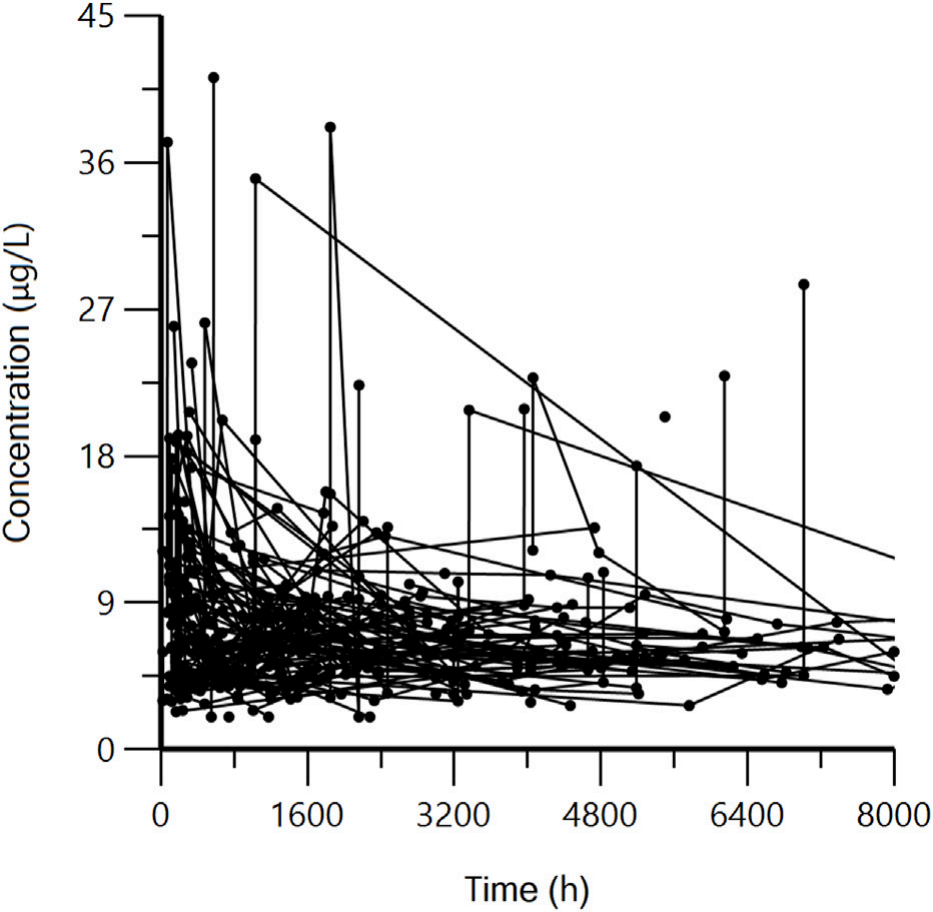
Tacrolimus concentration vs. time.

A total of 139 patients with 432 blood concentration data were included in this study, of which 35 were peak concentrations and the rest were trough concentrations. The curve of concentration varying with time is shown in Figure 1. All participants met the inclusion and exclusion criteria and signed an informed consent form. 139 patients had a median age and weight of 5.3 (range 1.1–15.6) years; 19.7 (range 9.5–88.0) kg, respectively, and a median blood concentration of 6.48 (range 2.0–41.2) μg/L. The final genotypes included in the PPK analysis were 12 SNPs, genotyping of 12 SNPs by genetic testing. All SNPs met Hardy-Weinberg equilibrium test. The clinical characteristics and genotypes of the patients are shown in Tables 1, 2, respectively.

**TABLE 1:** Demographic and clinical characteristics of patients.

Characteristics	Value (median, range)
Gender (male/female)	37/102
Age (years)	5.3 (1.1–15.6)
Weight (kg)	19.7 (9.5–88)
ALB (g/L)	18.3 (7.6–46.8)
HCT (%)	41.2 (23.4–447.6)
Combined use of wuzhi capsules (Yes/No)	6/133
Blood concentration (μg/L)	6.48 (2.0–41.2)

**TABLE 2:** Genotypes and allele frequencies of patients.

Genotypes	Number of patients	Percentage (%)	Allele	Allele frequency	H-W *p*-value
CYP3A5*3 rs 776746					
*1/*1	18	13.0	*1	0.338	0.727
*1/*3	58	41.7	*3	0.662	
*3/*3	63	45.3			
*MYH9* rs 2239781					
TT	52	37.4	T	0.604	0.908
TC	64	46.0	C	0.396	
CC	23	16.6			
*LAMB2* rs 62119873					
AA	73	52.5	A	0.727	0.986
AG	56	40.3	G	0.273	
GG	10	7.2			
*ACTN4* rs 62121818					
CC	27	19.4	C	0.414	0.532
CT	61	43.9	T	0.586	
TT	51	36.7			
*ACTN4* rs 3745859					
TT	28	20.1	T	0.417	0.416
TC	60	43.2	C	0.583	
CC	51	36.7			
*MAP3K11* rs 7946115					
CC	3	2.2	C	0.133	0.925
CG	31	22.3	G	0.867	
GG	105	75.5			
*ACTN4* rs 56113315					
TT	51	36.1	T	0.582	0.252
TC	60	44.0	C	0.418	
CC	28	19.9			
*MYH9* rs 4821478					
AA	11	7.9	A	0.233	0.273
AG	43	30.9	G	0.767	
GG	85	61.2			
*INF2* rs 1128880					
GG	3	2.2	G	0.108	0.477
GT	24	17.3	T	0.892	
TT	112	80.5			
*IL2RA* rs 12722489					
CC	110	79.1	C	0.885	0.628
CT	26	18.7	T	0.115	
TT	3	2.2			
*NPHS2* rs *2274622*					
CC	22	16.9	C	0.349	0.166
CT	53	63.2	T	0.651	
TT	64	58.9			
*CTLA4* rs 4553808					
GG	2	1.4	G	0.119	0.999
GA	29	20.9	A	0.881	
AA	108	77.7			

### Population pharmacokinetic modelling

A one-compartment model of first-order absorption and elimination was used to characterize the pharmacokinetics of TAC in pediatric patients with nephrotic syndrome. Exponential and proportional models were chosen to describe the interindividual variability and residual variability, respectively. The base model was $K_{a}\left(h^{-1}\right)=4.48,V\left(L\right)=144.52\times \exp\left(\eta V\right),\;CL\left(L/h\right)=7.05\times \exp\left(\eta CL\right)$ .

After a stepwise regression approach, covariates retained in the final model that significantly affected clearance included: age, co-administration of Wuzhi capsules, *CYP3A5* *3/*3 genotype, and *CTLA4* rs4553808 genotype. No covariates were screened that significantly influenced the apparent volume of distribution. The final model expression equations are shown in Eqs. 1, 2. The pharmacokinetic parameters of the final model were estimated in Table 3. The typical values of the population for CL and V were 10.54 L·h^−1^and 192.03 L, respectively.

V(L)=192.03×exp(ηV) (1)

$$CL\left(\frac{L}{h}\right)=10.54\times {\left(\frac{AGE}{5.3}\right)}^{0.31}\times \exp\left(-0.34\times \left(CTLA4\;GA\right)\right)\times \exp\left(-0.15\times \left(CTLA4\;AA\right)\right)\times \exp\left(-0.25\left(CYP3A5*3/*3\right)\right)\times \exp\left(-0.34\left(combWZ\right)\right)\times \exp\left(\eta CL\right)$$ (2)

(*CTLA4* GA = 1 and *CTLA4* AA = 1 when genotypes are *CTLA4* GA, *CTLA4* AA, respectively, 0 otherwise; *CYP3A5* *3/*3 = 1 when genotype is *CYP3A5* *3/*3, 0 otherwise; combWZ = 1 when combined with Wuzhi capsules, 0 otherwise.)

**TABLE 3:** Parameter estimation results and bootstrap results of the final population pharmacokinetic model.

Parameter	Final model	Bootstrap		
	Estimate (shrinkage %)	CV%	Median	95% CI
tvV(L)	192.03	18.76	192.03	126.72–289.71
vbtvCL (L/h)	10.54	6.81	10.38	7.55–14.21
dCLdAGE	0.31	12.95	0.31	0.22–0.39
dCLdCTLA4 GA	−0.34	−18.21	−0.33	−0.63 ∼ −0.01
dCldCTLA4 AA	−0.15	−28.96	−0.15	−0.43–0.18
dCLdCYP3A5 *3/*3	−0.25	−19.07	−0.25	−0.36 ∼ −0.15
dCLdCombWZ	−0.34	−25.96	−0.34	−0.52 ∼ −0.15
Inter-individual variability				
ω^2^_V_ (%)	1.13 (38.09)	5.53	1.13	—
ω^2^_CL_ (%)	0.13 (30.02)	6.03	0.13	—
Residual variability				
σ (%)	29.95	4.75	0.30	0.27–0.33

CV%, percent confidence of variation; CI, confidence interval; tvV, typical value of apparent distribution; tvCL, typical value of apparent volume clearance; dCLdAGE, fixed parameter coefficient of age to CL; dCLdCTLA4 GA, fixed parameter coefficient of *CTLA4* GA to CL; dCldCTLA4 AA, fixed parameter coefficient of *CTLA4* AA to CL; dCLdCYP3A5 *3/*3, fixed parameter coefficient of *CYP3A5* *3/*3 to CL; dCLdCombWZ, fixed parameter coefficient of WuZhi capsules to CL; ω^2^_V_, variance of interindividual variability for V; ω^2^_CL_, variance of interindividual variability for CL; σ, square root of residual variability for the final model.

### Model evaluation and validation

**FIGURE 2:**
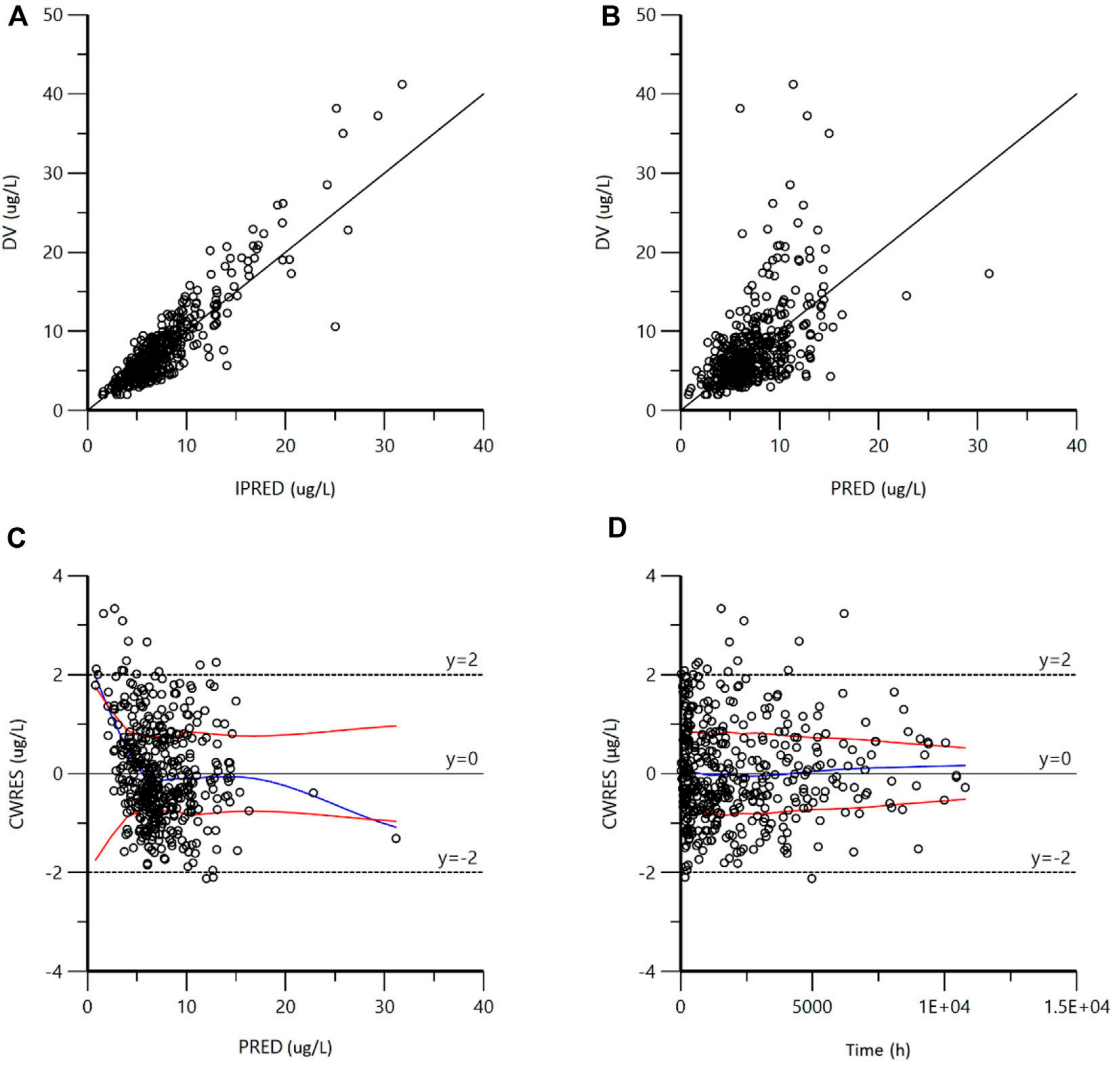
Goodness-of-fit plots for the final model. **(A)** Observed concentrations (DV) vs. individual predictions (IPRED); **(B)** DV vs. population predictions (PRED); **(C)** conditional weighted residuals (CWRES) vs. PRED; **(D)** CWRES vs. Time). As shown in figures **(A,B)**, the scatter points generated by the final model are evenly distributed on both sides of the reference line; as shown in figures C and D, the residual scatter points are symmetrically distributed. Most of them are in the range of (−2 ∼ + 2), which proves that there is no obvious bias in the prediction error.

The GOF plots of the final model are shown in Figure 2. The IPRED-DV scatterplot and IPRED-DV scatterplot show that the data in the final model are more evenly concentrated on both sides of the reference line, which proves that the prediction errors are not significantly biased. The CWRES-PRED scatterplot and CWRES-TIME scatterplot show that the residuals are more symmetrically distributed, and most of them are in the (y = −2 ∼ +2) range.

**FIGURE 3:**
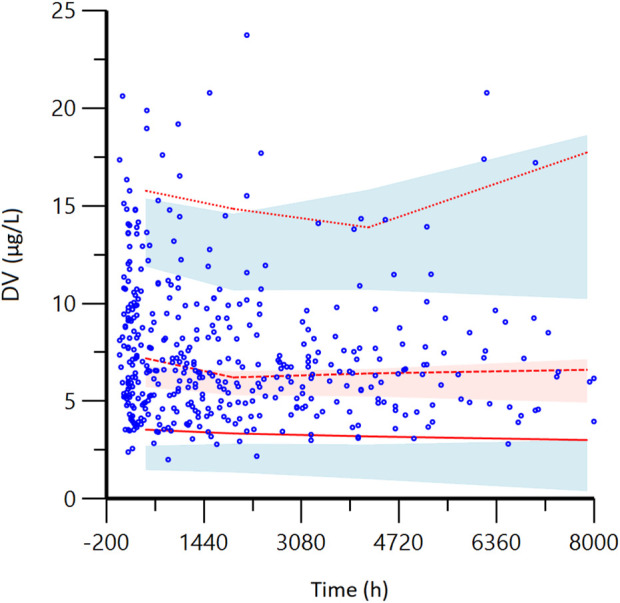
Visual predictive check (VPC) from the final model. DV: observed concentrations; The red lines from bottom to top represent the 5th, 50th, and 95th percentiles of the observed concentrations; Blue shaded regions are 95% confidence intervals for predicted 5th and 95th percentiles. Red shaded regions are 95% confidence intervals for the predicted 50th percentile. As shown in the figure, most of the red lines representing the measured concentration positioned are within the predicted shadow range, indicating that the model has good prediction performance.

The VPC results showed that the 95% confidence interval based on the simulation covered the 50% and 95% quartiles corresponding to the observed values. The observed values in the 5% quartile partially did not fall within the prediction interval, but the overall was closer to the prediction interval (<2.0 μg/L). This indicates that the final model has good predictive performance. See Figure 3.

**FIGURE 4:**
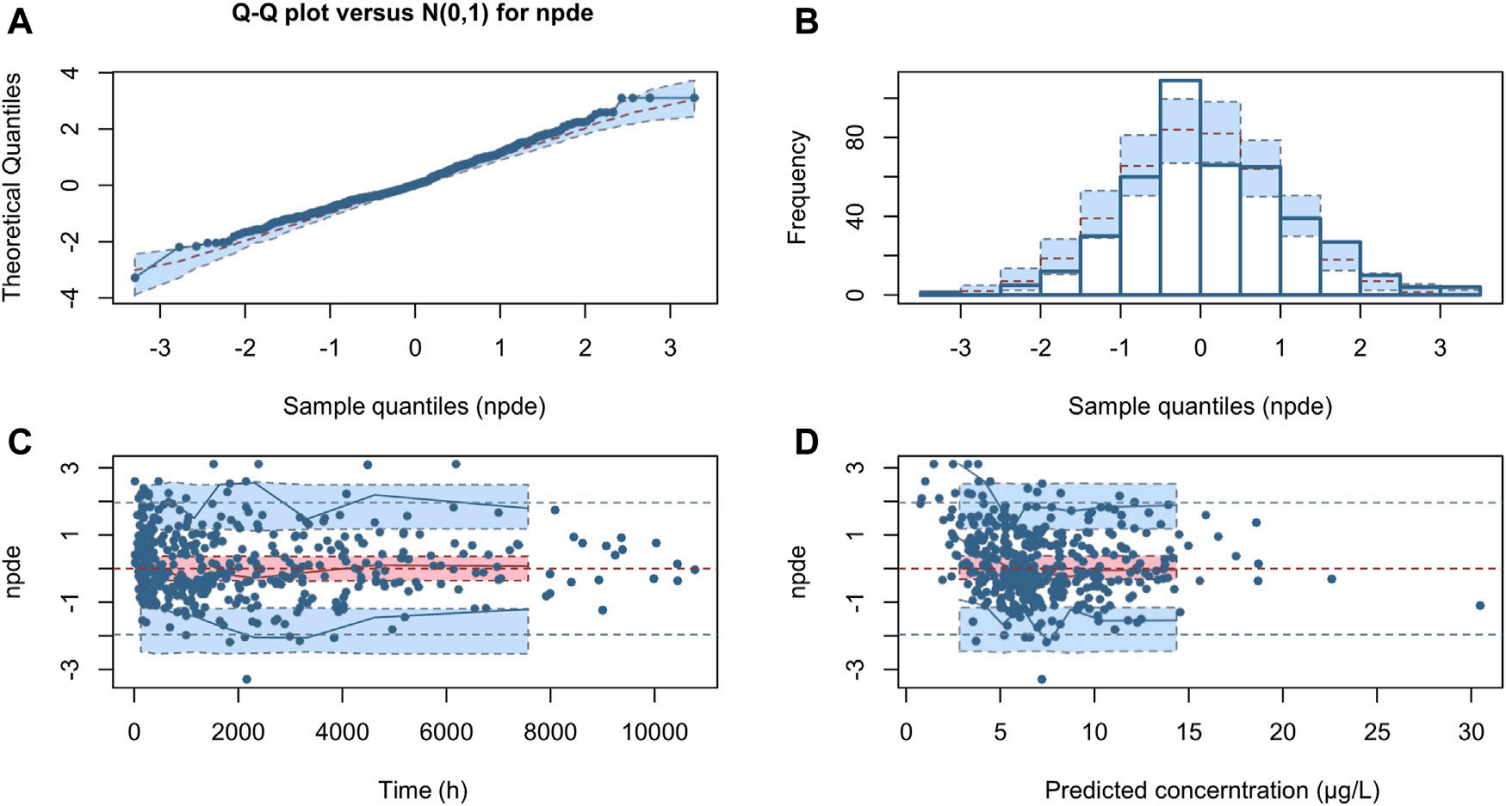
Normalized prediction distribution error (NPDE) for the final model. Quantile-quantile plots of NPDE vs. the expected standard normal distribution **(A)**. Histogram of NPDE values with the standard normal distribution overlayed **(B)**. Scatter plot of the time vs. NPDE **(C)**. Scatterplot of predictions vs. NPDE **(D)**.

The NPDE distribution and histogram are presented in Figure 4. The assumption of a normal distribution for the differences between predictions and observations was acceptable. The quantile-quantile plots and histogram also confirmed the normality of the NPDE, which indicates that the final model has strong predictive performance.

The comparison between the model parameters obtained from 1000 bootstrap validations and the final model parameters is shown in Table 3. The results show that the median parameter estimates obtained from the bootstrap process are consistent with the final model, and the final model parameters all fall within the 95% confidence interval of the bootstrap parameters, indicating that the final model has good robustness and accuracy.

### Machine learning model results and application

Based on the PPK model, we further used six machine learning algorithms, XGBoost, RF, Extra-Trees, GBDT, AdaBoost, and Lasso, to build a machine learning model on the clearance of TAC. Among the six machine learning models, the Lasso algorithm model has the largest R-squared (0.42), which is greater than the second-ranked Extra-Trees algorithm model (0.39). Other metrics include MSE, MAE, MedAE and RE, the Lasso algorithm model has the smallest value among all six models, indicating that the Lasso machine learning model is the optimal model. See Table 4. In clinical practice, we can input patients’ variables into the best model to predict the patient’s clearance rate, and then adjust the dose administered according to the target blood concentration.

**TABLE 4:** Algorithm performance results (test set).

Algorithm	R^2^	MSE	MAE	MedAE	RE
XGBoost	0.37	4.35	1.65	1.33	0.22
RF	0.34	4.57	1.68	1.40	0.24
Extra-Trees	0.39	4.17	1.58	1.22	0.22
GBDT	0.37	4.30	1.61	1.34	0.22
AdaBoost	0.38	4.25	1.63	1.30	0.23
Lasso	0.42	3.98	1.51	1.13	0.21

XGBoost: eXtreme Gradient Boosting, RF: Random Forest, GBDT: Gradient Boosting Decision Tree, AdaBoost: Adaptive boosting, MSE: Mean Square Error, MAE: Mean Absolute Error, MedAE: Median Absolute Error, RE: Root Mean Square Error.

## Discussion

In this study, a PPK model of TAC in a pediatric population with refractory nephrotic syndrome (RNS) was developed using a nonlinear mixed-effects model. Similar to most studies (Hao et al., 2018; Wang D. et al., 2019a; Wang X. et al., 2019b; Chen et al., 2020), the classical one-compartment model of first-order absorption and elimination adequately described the pharmacokinetic process of TAC, and the final model fit was good (Figures 2–4). The population typical value of the final model for estimating TAC clearance was 10.54 L h^−1^. Age, combined use of Wuzhi capsules, CYP3A5*3 rs776746 and *CTLA4* rs4553808 significantly affect TAC clearance. Based on this, six machine learning algorithms, XGBoost, RF, Extra-Trees, GBDT, AdaBoost, and Lasso, were used to build a machine learning model on TAC clearance, where the Lasso algorithm had the best model performance with the largest R^2^ value (0.42).

Unlike previous studies, we used PPK combined with machine learning for the first time to predict the individual clearance of TAC. Machine learning is an emerging and more advanced algorithm that is able to process complex, multidimensional, interacting variables for predictions by classification or regression. For example, machine learning has been used to screen genes affecting TAC pharmacokinetics (Gim et al., 2020) and to predict stable doses of TAC in renal transplant patients (Tang et al., 2017). However, machine learning has drawbacks, such as the inability to measure and distinguish intra- and inter-individual variability. The advantage of PPK to fully account for inter-and intra-individual variability and to quantify the effect of these variations on pharmacokinetic parameters precisely compensates for the shortcomings of machine learning (Bon et al., 2018; Campagne et al., 2019). Therefore, PPK combined with machine learning to accurately predict pharmacokinetics may be a better approach (van Gelder and Vinks, 2021; Yang et al., 2022). Tang et al. (2021) developed an individual clearance prediction model for neonatal renal clearance of drugs and successfully validated that PPK combined with machine learning can improve the prediction accuracy of drug clearance. In this study, we first obtained variables significantly associated with TAC clearance (age, combined use of Wuzhi capsules, CYP3A5*3 rs776746 and *CTLA4* rs4553808) by PPK approach, and then further developed a machine learning model for TAC clearance, and the final model had a good predictive performance with an R^2^ value of 0.42, similar to our previous tacrolimus dose/weight-adjusted trough concentration prediction model (R^2^ = 0.44), but superior to the groups of whether CYP3A5 was expressed (Mo et al., 2022); and the Lasso algorithm outperformed other machine learning algorithms such as XGBoost, RF, and Extra-Trees.

The final PPK model did not screen out covariates affecting the apparent volume of distribution of TAC, which may be related to the fact that we included mostly trough concentration data then not enough for the analysis of the distribution. The covariates associated with TAC clearance in the final model included: age, co-administration of wuzhi capsules, *CYP3A5* genotype, and *CTLA4* genotype (Table 3). Among them, age, wuzhi capsules, and *CYP3A5* genotype have been reported (Wang D. et al., 2019a; Chen et al., 2020). The results of this study showed that age affects the clearance of TAC, which may be related to the growth and development of children. Emoto et al. (2019) suggested that the age-dependent changes in TAC trough concentrations in pediatric patients were mainly attributable to the individual developmental characteristics of CYP3A. In addition, the individual clearance of TAC can be decreased by the combination of Wuzhi capsules. Studies have shown that the main active ingredients in Wuzhi capsules (pentosidine and pentosanol) can significantly increase TAC concentrations by inhibiting CYP3A and P-glycoprotein (P-gp)-mediated metabolism and transport (Qin et al., 2010; Wei et al., 2013; Qin et al., 2014; Cheng et al., 2021). In addition to age and combined use of Wuzhi capsules, *CYP3A5* genotype is an important influencing factor on the clearance of TAC. The Clinical Pharmacogenetics Implementation Consortium (CPIC) guideline recommendations for TAC use in transplant patients suggest that *CYP3A5* *3*3 carriers have a lower clearance, which means *CYP3A5* *3*3 carriers require a lower dose (Birdwell et al., 2015). This is also consistent with our findings that there is a negative correlation between *CYP3A5* *3*3 and clearance.

Among the numerous genetic variables included, in addition to the *CYP3A5* gene, the *CTLA4* gene also has a significant effect. CTLA4 (cytotoxic T lymphocyte-associated protein 4) is a receptor on the surface of T cells which is an inhibitory stimulator that inhibits T cell activation. Our results showed that the *CTLA4* rs4553808 significantly affected the clearance of TAC, The GA and AA genotypes showed a negative correlation with the clearance of TAC (Table 3). Similar to our findings, (Liu et al., 2017) found that the *CTLA4* rs4553808 genotype significantly affected the postoperative TAC concentration in Chinese kidney transplant patients. As is well known, CTLA4 is an inhibitory co-stimulatory factor in the CD28 family, competing with the T cell co-stimulatory receptor CD28 for the ligand B7-1 (CD80)/B7-2 (CD86) on the surface of antigen-presenting cells (Thompson and Allison, 1997; Crespo et al., 2013). This inhibits the activation of T cells and suppresses the secretion of the cytokine interleukin 2 (IL2). Similarly, the complex formed by TAC and FK-binding protein 12 (FKBP12) can block the production of IL2 (Li and Li, 2015; Martial et al., 2021). Therefore, CTLA4 may act as an upstream regulator of IL2 secretion and indirectly influence the pharmacokinetic process of TAC by affecting IL2 production. In addition, some *in vitro* experiments have shown that increased inflammatory protein production by hepatocytes during the inflammatory response reduces the ability of hepatocytes to metabolize drugs through the cytochrome P450 system (Abdel-Razzak et al., 1993; Elkahwaji et al., 1999; Ferri et al., 2016). IL2, one of the important inflammatory cytokines, decreased the activity of CYP3A4 by 39% (Elkahwaji et al., 1999). Therefore, polymorphisms in the *CTLA4* gene may affect the individual clearance of TAC by influencing the concentration of IL2 and thus the activity of TAC CYP3A4 metabolizing enzymes. In addition to this, there are also relevant studies suggesting that the pathogenesis of nephrotic syndrome may be related to CTLA4-mediated T-cell dysfunction (Reiser et al., 2004; Yu et al., 2013). Therefore, the effect of CTLA4 on TAC in the nephrotic syndrome population deserves to be explored in depth.

Our study also has some limitations. First, most of the blood samples collected were trough concentration points, and only a few had other points such as peak concentration points. This causes the performance of the model still needs to be improved, and also causes the present model less predictive at low concentrations. Second, the covariates we included can still be expanded, we currently included only clinical characteristics of patients and genomic data. In the future, more bioinformatics data, such as metabolome, proteome, gut microbiome and other multi-omics data, need to be incorporated by using machine learning to improve the prediction performance of TAC pharmacokinetics.

## Conclusion

In summary, we firstly used PPK combined with machine learning to develop a precise model of TAC clearance in pediatric patients with nephrotic syndrome from the perspective of clinical characteristics and genetic polymorphisms. Age, combined use of Wuzhi capsules, CYP3A5*3 rs776746 covariates and *CTLA4* rs4553808 were found to significantly affect the pharmacokinetics of TAC. For the first time, *CTLA4* rs4553808 gene polymorphism was found to affect the clearance of TAC in the nephrotic syndrome population. The model can predict the individual clearance of TAC more accurately. It provides a reference for the clinical pharmacist or clinician to optimize the initial dose administered, ensuring the effectiveness and safety of drug treatment for each patient.

## Data Availability

The original contributions presented in the study are included in the article, further inquiries can be directed to the corresponding authors.
